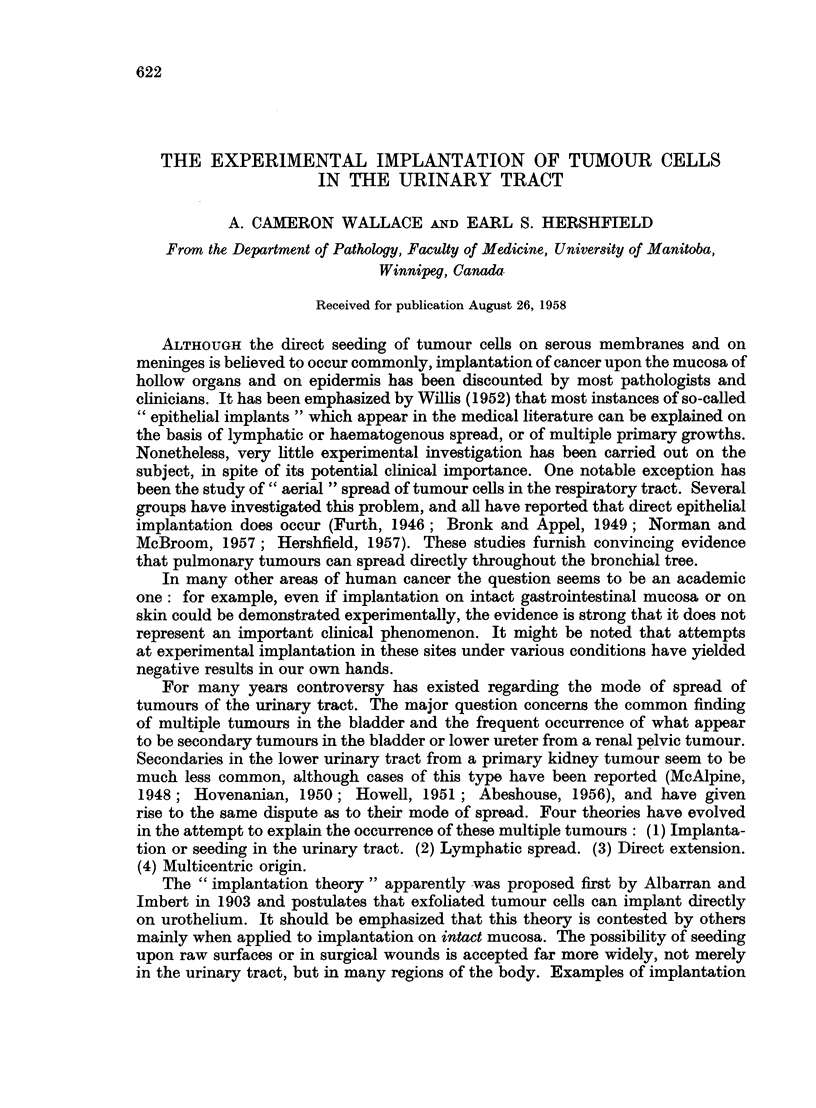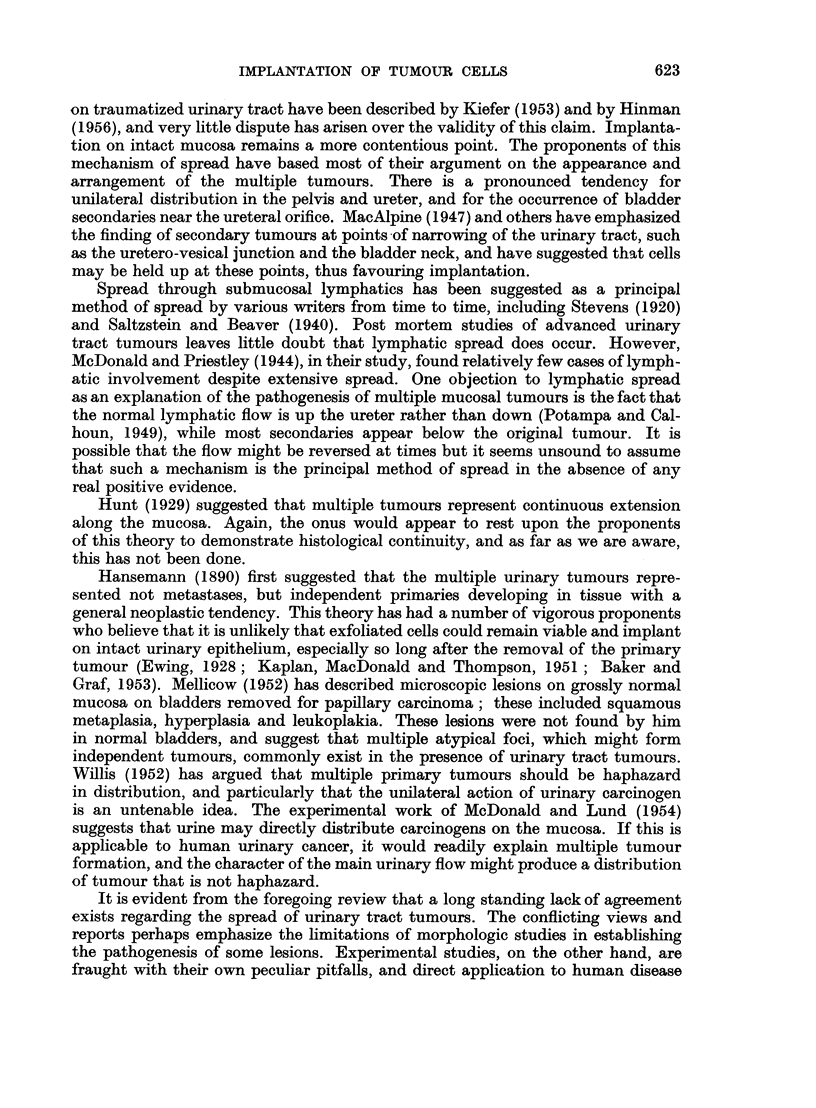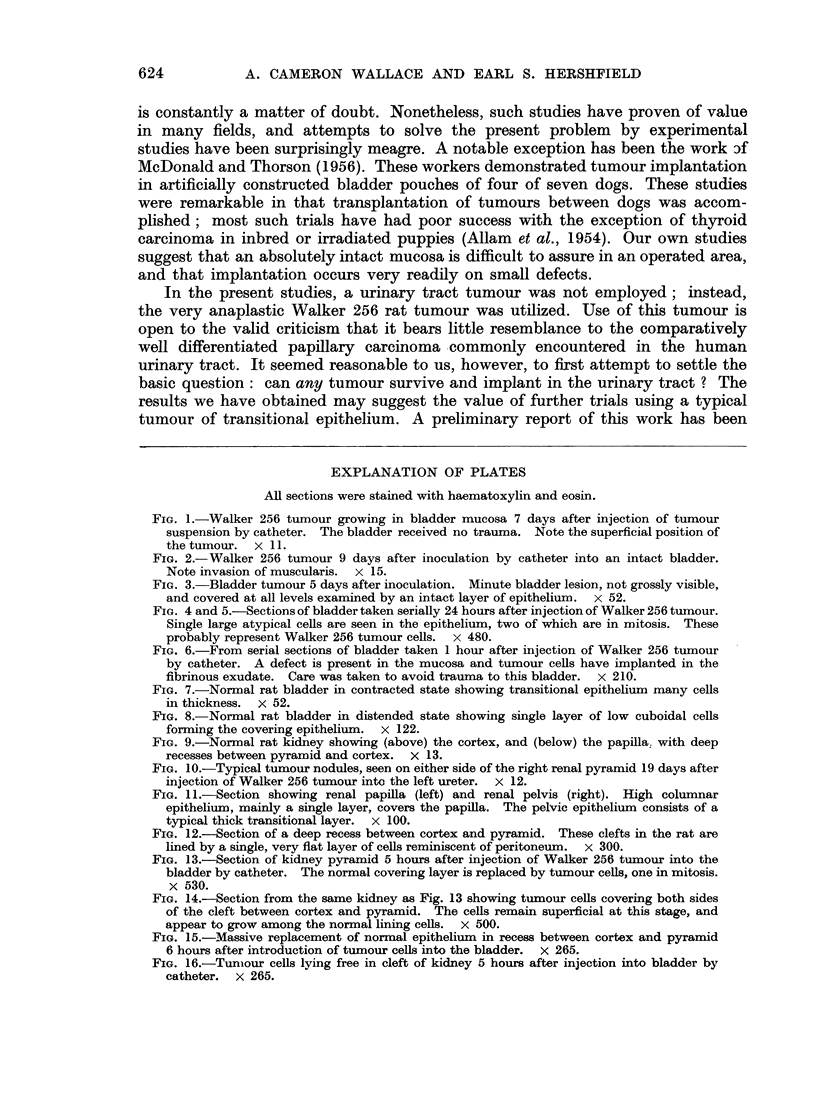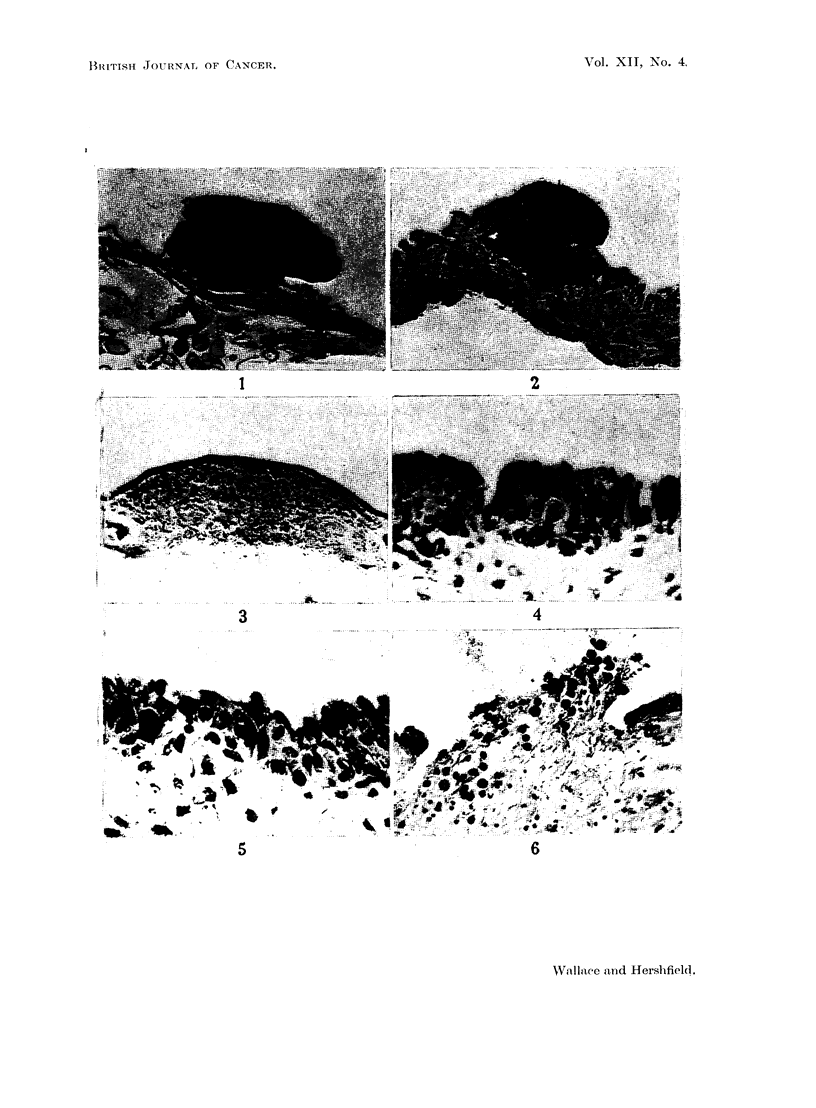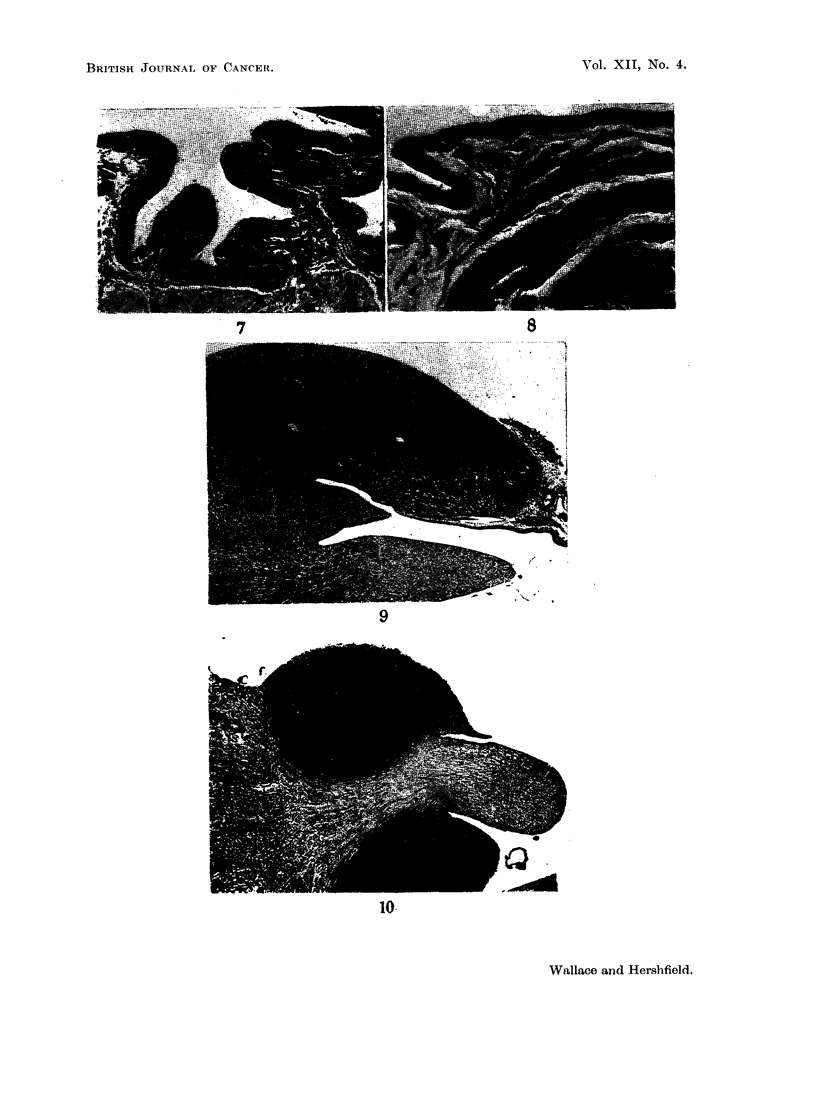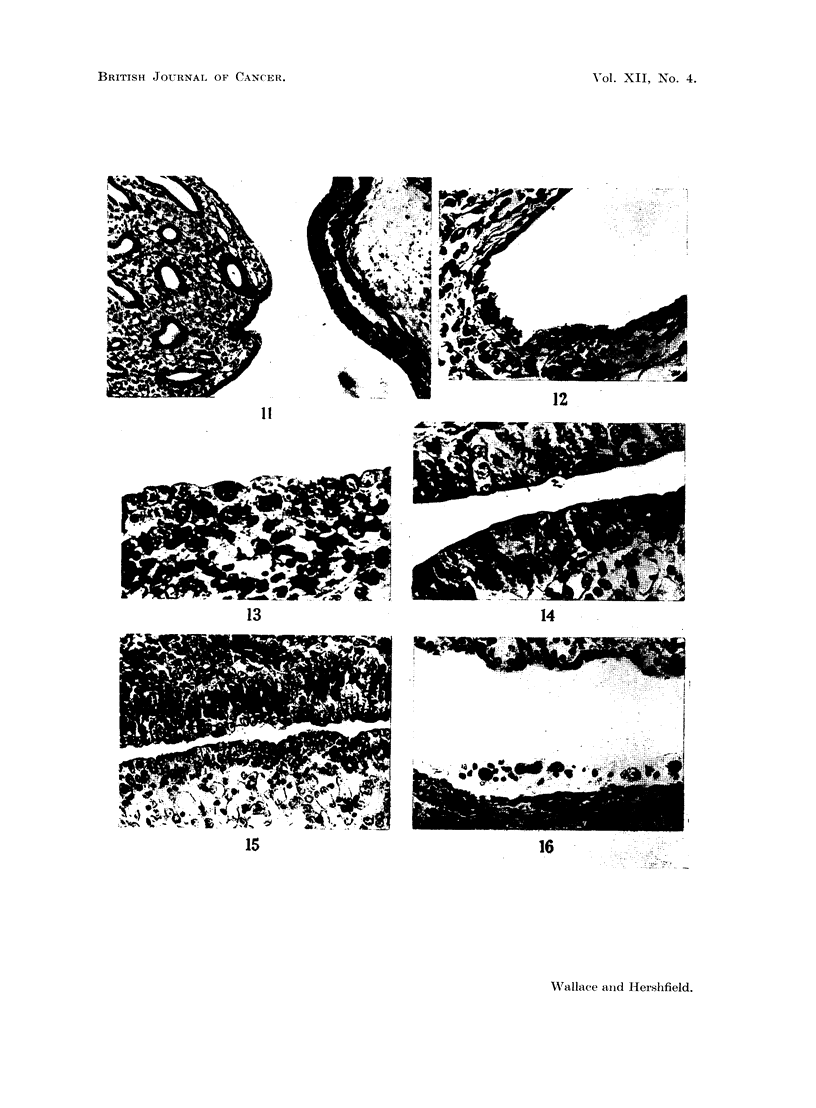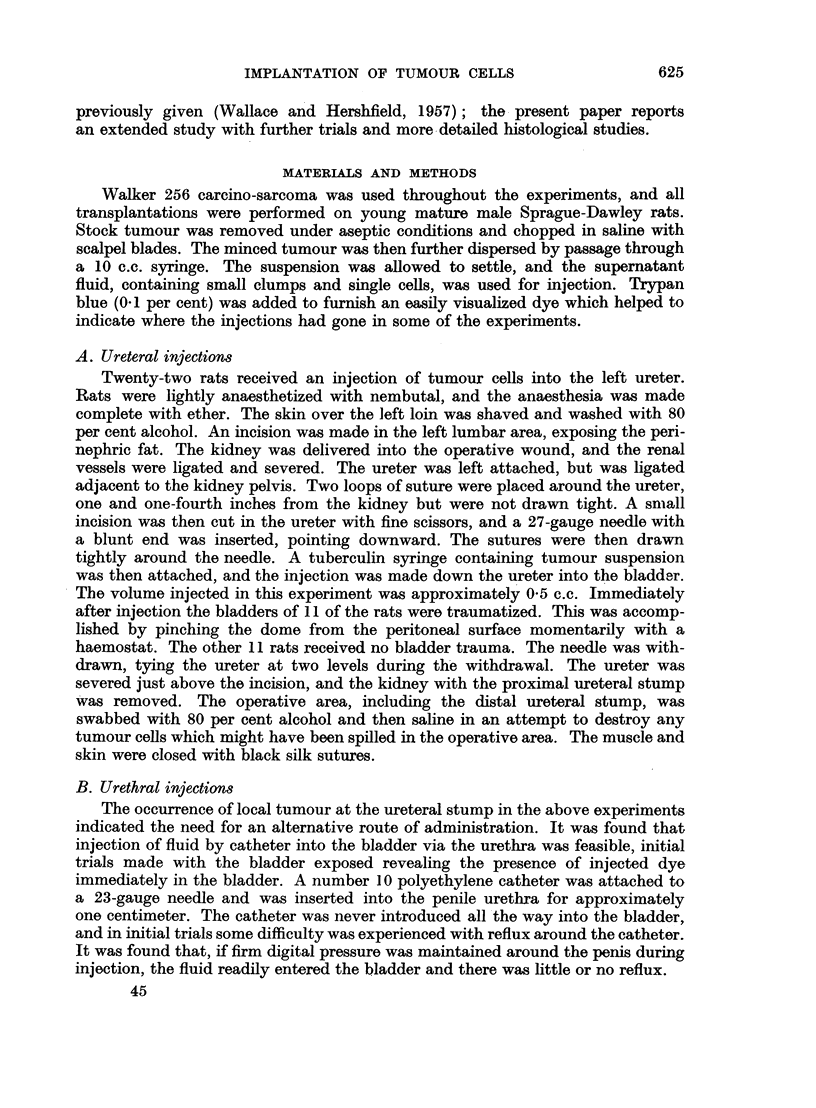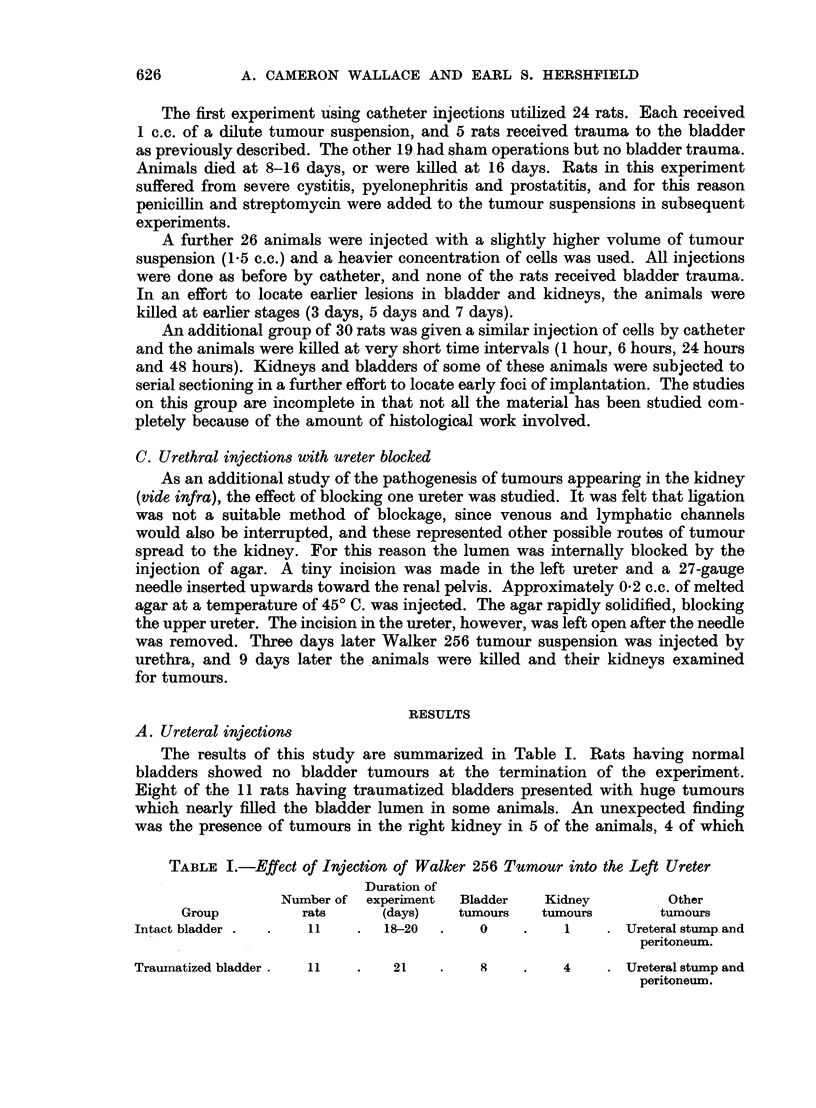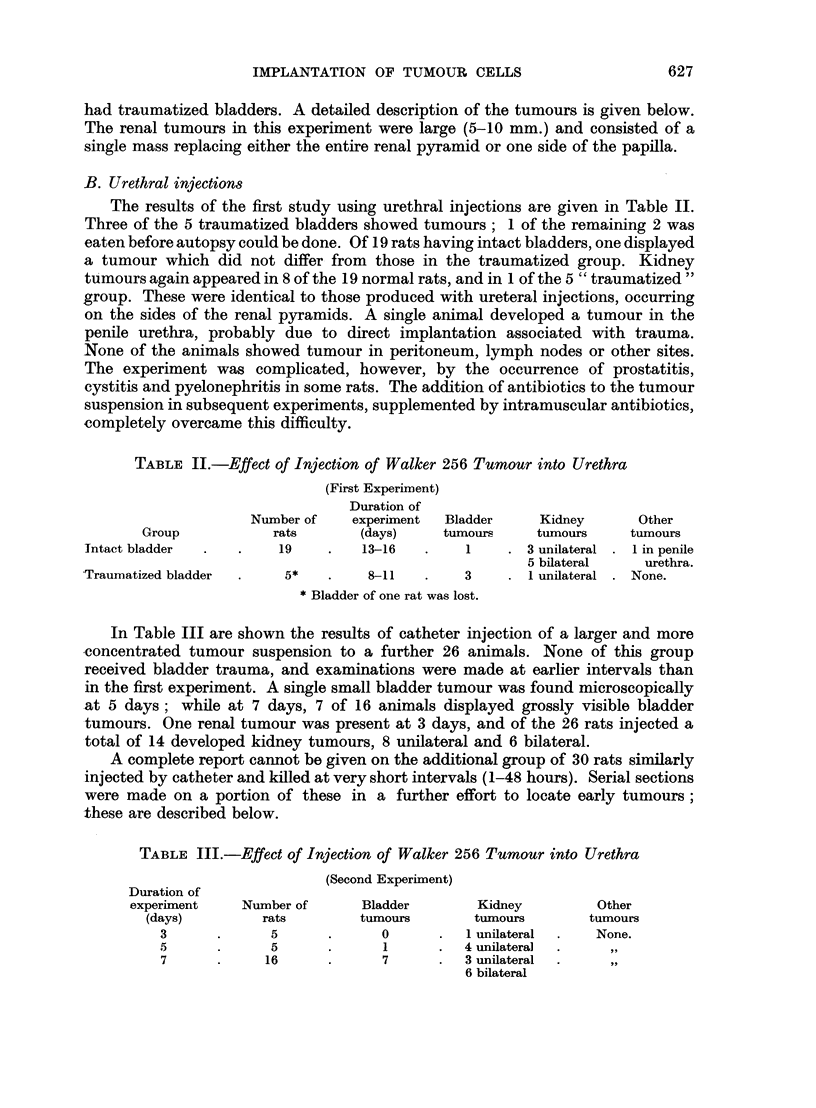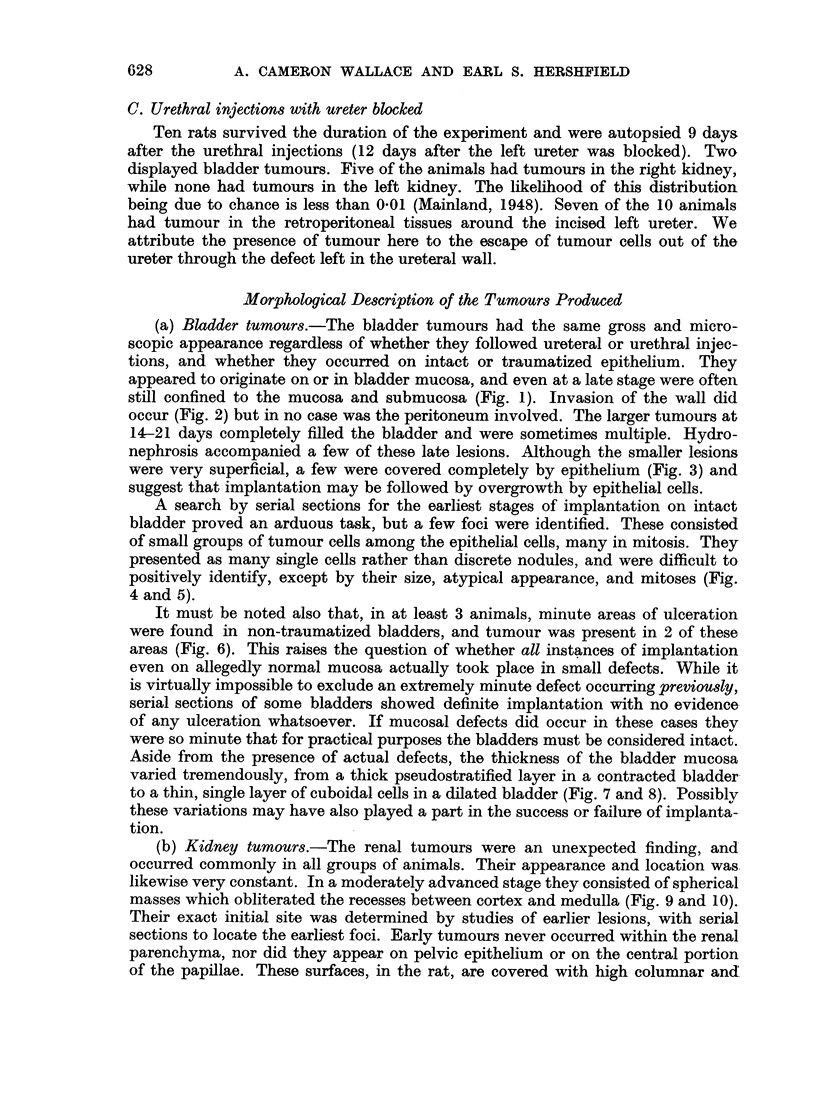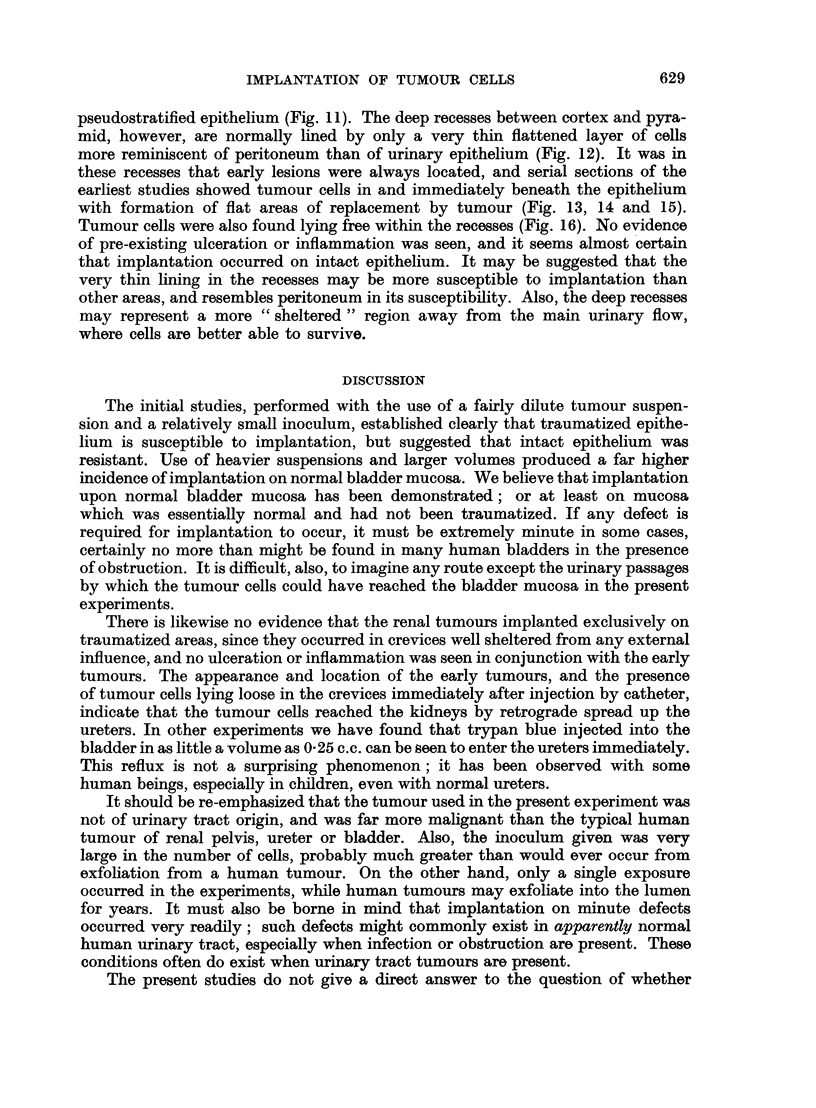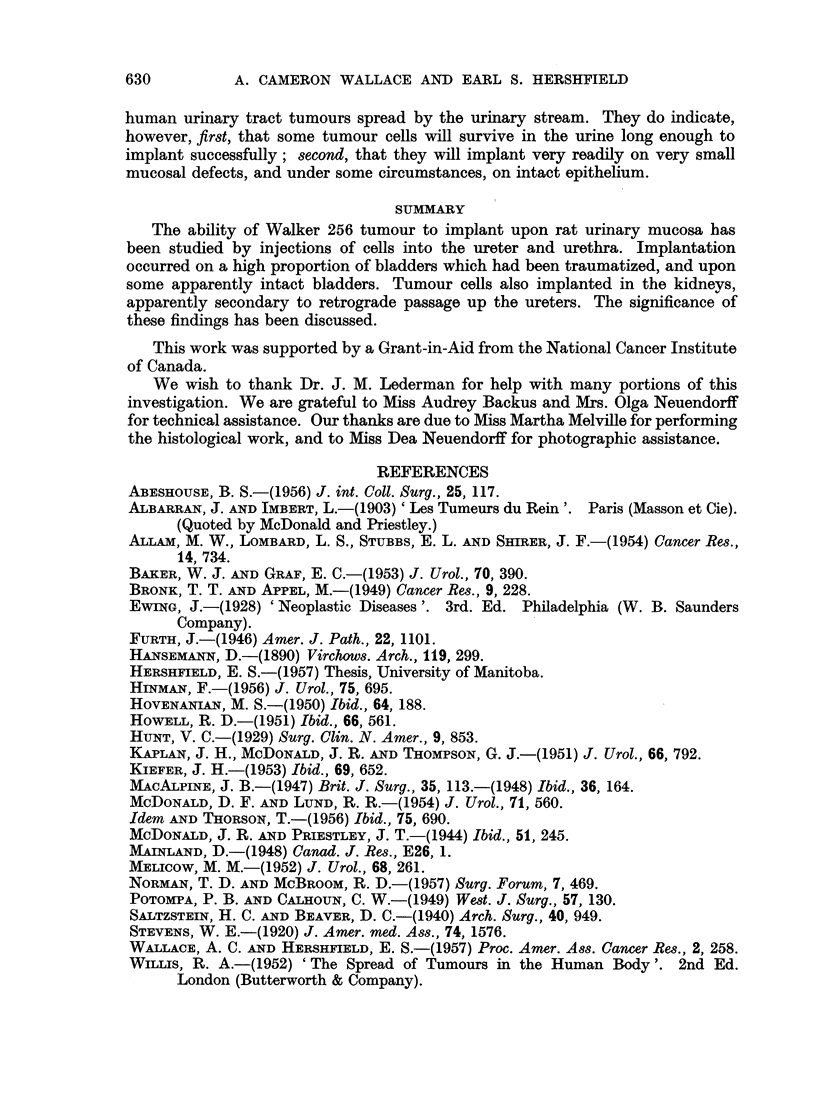# The Experimental Implantation of Tumour Cells in the Urinary Tract

**DOI:** 10.1038/bjc.1958.70

**Published:** 1958-12

**Authors:** A. Cameron Wallace, Earl S. Hershfield

## Abstract

**Images:**


### THE EXPERIMENTAL IMPLANTATION OF TUMOUR CELLS IN THE URINARY TRACT

A. CAMERON WALLACE AND EARL S. HERSHFIELD

*From the Department of Pathology, Faculty of Medicine, University of Manitoba, Winnipeg, Canada*

Received for publication August 26, 1958

ALTHOUGH the direct seeding of tumour cells on serous membranes and on meninges is believed to occur commonly, implantation of cancer upon the mucosa of hollow organs and on epidermis has been discounted by most pathologists and clinicians. It has been emphasized by Willis (1952) that most instances of so-called " epithelial implants " which appear in the medical literature can be explained on the basis of lymphatic or haematogenous spread, or of multiple primary growths. Nonetheless, very little experimental investigation has been carried out on the subject, in spite of its potential clinical importance. One notable exception has been the study of " aerial " spread of tumour cells in the respiratory tract. Several groups have investigated this problem, and all have reported that direct epithelial implantation does occur (Furth, 1946 ; Bronk and Appel, 1949 ; Norman and McBroom, 1957 ; Hershfield, 1957). These studies furnish convincing evidence that pulmonary tumours can spread directly throughout the bronchial tree.

In many other areas of human cancer the question seems to be an academic one : for example, even if implantation on intact gastrointestinal mucosa or on skin could be demonstrated experimentally, the evidence is strong that it does not represent an important clinical phenomenon. It might be noted that attempts at experimental implantation in these sites under various conditions have yielded negative results in our own hands.

For many years controversy has existed regarding the mode of spread of tumours of the urinary tract. The major question concerns the common finding of multiple tumours in the bladder and the frequent occurrence of what appear to be secondary tumours in the bladder or lower ureter from a renal pelvic tumour. Secondaries in the lower urinary tract from a primary kidney tumour seem to be much less common, although cases of this type have been reported (McAlpine, 1948 ; Hovenanian, 1950 ; Howell, 1951 ; Abeshouse, 1956), and have given rise to the same dispute as to their mode of spread. Four theories have evolved in the attempt to explain the occurrence of these multiple tumours : (1) Implantation or seeding in the urinary tract. (2) Lymphatic spread. (3) Direct extension. (4) Multicentric origin.

The " implantation theory " apparently was proposed first by Albarran and Imbert in 1903 and postulates that exfoliated tumour cells can implant directly on urothelium. It should be emphasized that this theory is contested by others mainly when applied to implantation on *intact* mucosa. The possibility of seeding upon raw surfaces or in surgical wounds is accepted far more widely, not merely in the urinary tract, but in many regions of the body. Examples of implantation on traumatized urinary tract have been described by Kiefer (1953) and by Hinman (1956), and very little dispute has arisen over the validity of this claim. Implantation on intact mucosa remains a more contentious point. The proponents of this mechanism of spread have based most of their argument on the appearance and arrangement of the multiple tumours. There is a pronounced tendency for unilateral distribution in the pelvis and ureter, and for the occurrence of bladder secondaries near the ureteral orifice. MacAlpine (1947) and others have emphasized the finding of secondary tumours at points of narrowing of the urinary tract, such as the uretero-vesical junction and the bladder neck, and have suggested that cells may be held up at these points, thus favouring implantation.

Spread through submucosal lymphatics has been suggested as a principal method of spread by various writers from time to time, including Stevens (1920) and Saltzstein and Beaver (1940). Post mortem studies of advanced urinary tract tumours leaves little doubt that lymphatic spread does occur. However, McDonald and Priestley (1944), in their study, found relatively few cases of lymphatic involvement despite extensive spread. One objection to lymphatic spread as an explanation of the pathogenesis of multiple mucosal tumours is the fact that the normal lymphatic flow is up the ureter rather than down (Potampa and Calhoun, 1949), while most secondaries appear below the original tumour. It is possible that the flow might be reversed at times but it seems unsound to assume that such a mechanism is the principal method of spread in the absence of any real positive evidence.

Hunt (1929) suggested that multiple tumours represent continuous extension along the mucosa. Again, the onus would appear to rest upon the proponents of this theory to demonstrate histological continuity, and as far as we are aware, this has not been done.

Hansemann (1890) first suggested that the multiple urinary tumours represented not metastases, but independent primaries developing in tissue with a general neoplastic tendency. This theory has had a number of vigorous proponents who believe that it is unlikely that exfoliated cells could remain viable and implant on intact urinary epithelium, especially so long after the removal of the primary tumour (Ewing, 1928 ; Kaplan, MacDonald and Thompson, 1951 ; Baker and Graf, 1953). Mellicow (1952) has described microscopic lesions on grossly normal mucosa on bladders removed for papillary carcinoma ; these included squamous metaplasia, hyperplasia and leukoplakia. These lesions were not found by him in normal bladders, and suggest that multiple atypical foci, which might form independent tumours, commonly exist in the presence of urinary tract tumours. Willis (1952) has argued that multiple primary tumours should be haphazard in distribution, and particularly that the unilateral action of urinary carcinogen is an untenable idea. The experimental work of McDonald and Lund (1954) suggests that urine may directly distribute carcinogens on the mucosa. If this is applicable to human urinary cancer, it would readily explain multiple tumour formation, and the character of the main urinary flow might produce a distribution of tumour that is not haphazard.

It is evident from the foregoing review that a long standing lack of agreement exists regarding the spread of urinary tract tumours. The conflicting views and reports perhaps emphasize the limitations of morphologic studies in establishing the pathogenesis of some lesions. Experimental studies, on the other hand, are fraught with their own peculiar pitfalls, and direct application to human disease is constantly a matter of doubt. Nonetheless, such studies have proven of value in many fields, and attempts to solve the present problem by experimental studies have been surprisingly meagre. A notable exception has been the work of McDonald and Thorson (1956). These workers demonstrated tumour implantation in artificially constructed bladder pouches of four of seven dogs. These studies were remarkable in that transplantation of tumours between dogs was accomplished; most such trials have had poor success with the exception of thyroid carcinoma in inbred or irradiated puppies (Allam *et al.*, 1954). Our own studies suggest that an absolutely intact mucosa is difficult to assure in an operated area, and that implantation occurs very readily on small defects.

In the present studies, a urinary tract tumour was not employed; instead, the very anaplastic Walker 256 rat tumour was utilized. Use of this tumour is open to the valid criticism that it bears little resemblance to the comparatively well differentiated papillary carcinoma commonly encountered in the human urinary tract. It seemed reasonable to us, however, to first attempt to settle the basic question: can *any* tumour survive and implant in the urinary tract? The results we have obtained may suggest the value of further trials using a typical tumour of transitional epithelium. A preliminary report of this work has been

---

EXPLANATION OF PLATES

All sections were stained with haematoxylin and eosin.

FIG. 1.—Walker 256 tumour growing in bladder mucosa 7 days after injection of tumour suspension by catheter. The bladder received no trauma. Note the superficial position of the tumour. × 11.

FIG. 2.—Walker 256 tumour 9 days after inoculation by catheter into an intact bladder. Note invasion of muscularis. × 15.

FIG. 3.—Bladder tumour 5 days after inoculation. Minute bladder lesion, not grossly visible, and covered at all levels examined by an intact layer of epithelium. × 52.

FIG. 4 and 5.—Sections of bladder taken serially 24 hours after injection of Walker 256 tumour. Single large atypical cells are seen in the epithelium, two of which are in mitosis. These probably represent Walker 256 tumour cells. × 480.

FIG. 6.—From serial sections of bladder taken 1 hour after injection of Walker 256 tumour by catheter. A defect is present in the mucosa and tumour cells have implanted in the fibrinous exudate. Care was taken to avoid trauma to this bladder. × 210.

FIG. 7.—Normal rat bladder in contracted state showing transitional epithelium many cells in thickness. × 52.

FIG. 8.—Normal rat bladder in distended state showing single layer of low cuboidal cells forming the covering epithelium. × 122.

FIG. 9.—Normal rat kidney showing (above) the cortex, and (below) the papilla, with deep recesses between pyramid and cortex. × 13.

FIG. 10.—Typical tumour nodules, seen on either side of the right renal pyramid 19 days after injection of Walker 256 tumour into the left ureter. × 12.

FIG. 11.—Section showing renal papilla (left) and renal pelvis (right). High columnar epithelium, mainly a single layer, covers the papilla. The pelvic epithelium consists of a typical thick transitional layer. × 100.

FIG. 12.—Section of a deep recess between cortex and pyramid. These clefts in the rat are lined by a single, very flat layer of cells reminiscent of peritoneum. × 300.

FIG. 13.—Section of kidney pyramid 5 hours after injection of Walker 256 tumour into the bladder by catheter. The normal covering layer is replaced by tumour cells, one in mitosis. × 530.

FIG. 14.—Section from the same kidney as Fig. 13 showing tumour cells covering both sides of the cleft between cortex and pyramid. The cells remain superficial at this stage, and appear to grow among the normal lining cells. × 500.

FIG. 15.—Massive replacement of normal epithelium in recess between cortex and pyramid 6 hours after introduction of tumour cells into the bladder. × 265.

FIG. 16.—Tumour cells lying free in cleft of kidney 5 hours after injection into bladder by catheter. × 265.

1 2 3 4 5 6

Wallace and Hershfield.

7

8

9

10

Wallace and Hershfield.

11

12

13

14

15

16

Wallace and Hershfield.

previously given (Wallace and Hershfield, 1957); the present paper reports an extended study with further trials and more detailed histological studies.

### MATERIALS AND METHODS

Walker 256 carcino-sarcoma was used throughout the experiments, and all transplantations were performed on young mature male Sprague-Dawley rats. Stock tumour was removed under aseptic conditions and chopped in saline with scalpel blades. The minced tumour was then further dispersed by passage through a 10 c.c. syringe. The suspension was allowed to settle, and the supernatant fluid, containing small clumps and single cells, was used for injection. Trypan blue (0·1 per cent) was added to furnish an easily visualized dye which helped to indicate where the injections had gone in some of the experiments.

### *A. Ureteral injections*

Twenty-two rats received an injection of tumour cells into the left ureter. Rats were lightly anaesthetized with nembutal, and the anaesthesia was made complete with ether. The skin over the left loin was shaved and washed with 80 per cent alcohol. An incision was made in the left lumbar area, exposing the perinephric fat. The kidney was delivered into the operative wound, and the renal vessels were ligated and severed. The ureter was left attached, but was ligated adjacent to the kidney pelvis. Two loops of suture were placed around the ureter, one and one-fourth inches from the kidney but were not drawn tight. A small incision was then cut in the ureter with fine scissors, and a 27-gauge needle with a blunt end was inserted, pointing downward. The sutures were then drawn tightly around the needle. A tuberculin syringe containing tumour suspension was then attached, and the injection was made down the ureter into the bladder. The volume injected in this experiment was approximately 0·5 c.c. Immediately after injection the bladders of 11 of the rats were traumatized. This was accomplished by pinching the dome from the peritoneal surface momentarily with a haemostat. The other 11 rats received no bladder trauma. The needle was withdrawn, tying the ureter at two levels during the withdrawal. The ureter was severed just above the incision, and the kidney with the proximal ureteral stump was removed. The operative area, including the distal ureteral stump, was swabbed with 80 per cent alcohol and then saline in an attempt to destroy any tumour cells which might have been spilled in the operative area. The muscle and skin were closed with black silk sutures.

### *B. Urethral injections*

The occurrence of local tumour at the ureteral stump in the above experiments indicated the need for an alternative route of administration. It was found that injection of fluid by catheter into the bladder via the urethra was feasible, initial trials made with the bladder exposed revealing the presence of injected dye immediately in the bladder. A number 10 polyethylene catheter was attached to a 23-gauge needle and was inserted into the penile urethra for approximately one centimeter. The catheter was never introduced all the way into the bladder, and in initial trials some difficulty was experienced with reflux around the catheter. It was found that, if firm digital pressure was maintained around the penis during injection, the fluid readily entered the bladder and there was little or no reflux.

The first experiment using catheter injections utilized 24 rats. Each received 1 c.c. of a dilute tumour suspension, and 5 rats received trauma to the bladder as previously described. The other 19 had sham operations but no bladder trauma. Animals died at 8–16 days, or were killed at 16 days. Rats in this experiment suffered from severe cystitis, pyelonephritis and prostatitis, and for this reason penicillin and streptomycin were added to the tumour suspensions in subsequent experiments.

A further 26 animals were injected with a slightly higher volume of tumour suspension (1·5 c.c.) and a heavier concentration of cells was used. All injections were done as before by catheter, and none of the rats received bladder trauma. In an effort to locate earlier lesions in bladder and kidneys, the animals were killed at earlier stages (3 days, 5 days and 7 days).

An additional group of 30 rats was given a similar injection of cells by catheter and the animals were killed at very short time intervals (1 hour, 6 hours, 24 hours and 48 hours). Kidneys and bladders of some of these animals were subjected to serial sectioning in a further effort to locate early foci of implantation. The studies on this group are incomplete in that not all the material has been studied completely because of the amount of histological work involved.

*C. Urethral injections with ureter blocked*

As an additional study of the pathogenesis of tumours appearing in the kidney (*vide infra*), the effect of blocking one ureter was studied. It was felt that ligation was not a suitable method of blockage, since venous and lymphatic channels would also be interrupted, and these represented other possible routes of tumour spread to the kidney. For this reason the lumen was internally blocked by the injection of agar. A tiny incision was made in the left ureter and a 27-gauge needle inserted upwards toward the renal pelvis. Approximately 0·2 c.c. of melted agar at a temperature of 45° C. was injected. The agar rapidly solidified, blocking the upper ureter. The incision in the ureter, however, was left open after the needle was removed. Three days later Walker 256 tumour suspension was injected by urethra, and 9 days later the animals were killed and their kidneys examined for tumours.

### RESULTS

*A. Ureteral injections*

The results of this study are summarized in Table I. Rats having normal bladders showed no bladder tumours at the termination of the experiment. Eight of the 11 rats having traumatized bladders presented with huge tumours which nearly filled the bladder lumen in some animals. An unexpected finding was the presence of tumours in the right kidney in 5 of the animals, 4 of which

TABLE I.—*Effect of Injection of Walker 256 Tumour into the Left Ureter*

| Group | Number of rats | Duration of experiment (days) | Bladder tumours | Kidney tumours | Other tumours |
|---|---|---|---|---|---|
| Intact bladder | 11 | 18–20 | 0 | 1 | Ureteral stump and peritoneum. |
| Traumatized bladder | 11 | 21 | 8 | 4 | Ureteral stump and peritoneum. |

had traumatized bladders. A detailed description of the tumours is given below. The renal tumours in this experiment were large (5–10 mm.) and consisted of a single mass replacing either the entire renal pyramid or one side of the papilla.

*B. Urethral injections*

The results of the first study using urethral injections are given in Table II. Three of the 5 traumatized bladders showed tumours ; 1 of the remaining 2 was eaten before autopsy could be done. Of 19 rats having intact bladders, one displayed a tumour which did not differ from those in the traumatized group. Kidney tumours again appeared in 8 of the 19 normal rats, and in 1 of the 5 " traumatized " group. These were identical to those produced with ureteral injections, occurring on the sides of the renal pyramids. A single animal developed a tumour in the penile urethra, probably due to direct implantation associated with trauma. None of the animals showed tumour in peritoneum, lymph nodes or other sites. The experiment was complicated, however, by the occurrence of prostatitis, cystitis and pyelonephritis in some rats. The addition of antibiotics to the tumour suspension in subsequent experiments, supplemented by intramuscular antibiotics, completely overcame this difficulty.

TABLE II.—*Effect of Injection of Walker 256 Tumour into Urethra*

(First Experiment)

| Group | Number of rats | Duration of experiment (days) | Bladder tumours | Kidney tumours | Other tumours |
|---|---|---|---|---|---|
| Intact bladder | 19 | 13–16 | 1 | 3 unilateral<br>5 bilateral | 1 in penile urethra. |
| Traumatized bladder | 5* | 8–11 | 3 | 1 unilateral | None. |

* Bladder of one rat was lost.

In Table III are shown the results of catheter injection of a larger and more concentrated tumour suspension to a further 26 animals. None of this group received bladder trauma, and examinations were made at earlier intervals than in the first experiment. A single small bladder tumour was found microscopically at 5 days ; while at 7 days, 7 of 16 animals displayed grossly visible bladder tumours. One renal tumour was present at 3 days, and of the 26 rats injected a total of 14 developed kidney tumours, 8 unilateral and 6 bilateral.

A complete report cannot be given on the additional group of 30 rats similarly injected by catheter and killed at very short intervals (1–48 hours). Serial sections were made on a portion of these in a further effort to locate early tumours ; these are described below.

TABLE III.—*Effect of Injection of Walker 256 Tumour into Urethra*

(Second Experiment)

| Duration of experiment (days) | Number of rats | Bladder tumours | Kidney tumours | Other tumours |
|---|---|---|---|---|
| 3 | 5 | 0 | 1 unilateral | None. |
| 5 | 5 | 1 | 4 unilateral | ,, |
| 7 | 16 | 7 | 3 unilateral<br>6 bilateral | ,, |

*C. Urethral injections with ureter blocked*

Ten rats survived the duration of the experiment and were autopsied 9 days after the urethral injections (12 days after the left ureter was blocked). Two displayed bladder tumours. Five of the animals had tumours in the right kidney, while none had tumours in the left kidney. The likelihood of this distribution being due to chance is less than 0·01 (Mainland, 1948). Seven of the 10 animals had tumour in the retroperitoneal tissues around the incised left ureter. We attribute the presence of tumour here to the escape of tumour cells out of the ureter through the defect left in the ureteral wall.

### *Morphological Description of the Tumours Produced*

(a) *Bladder tumours.*—The bladder tumours had the same gross and microscopic appearance regardless of whether they followed ureteral or urethral injections, and whether they occurred on intact or traumatized epithelium. They appeared to originate on or in bladder mucosa, and even at a late stage were often still confined to the mucosa and submucosa (Fig. 1). Invasion of the wall did occur (Fig. 2) but in no case was the peritoneum involved. The larger tumours at 14–21 days completely filled the bladder and were sometimes multiple. Hydronephrosis accompanied a few of these late lesions. Although the smaller lesions were very superficial, a few were covered completely by epithelium (Fig. 3) and suggest that implantation may be followed by overgrowth by epithelial cells.

A search by serial sections for the earliest stages of implantation on intact bladder proved an arduous task, but a few foci were identified. These consisted of small groups of tumour cells among the epithelial cells, many in mitosis. They presented as many single cells rather than discrete nodules, and were difficult to positively identify, except by their size, atypical appearance, and mitoses (Fig. 4 and 5).

It must be noted also that, in at least 3 animals, minute areas of ulceration were found in non-traumatized bladders, and tumour was present in 2 of these areas (Fig. 6). This raises the question of whether *all* instances of implantation even on allegedly normal mucosa actually took place in small defects. While it is virtually impossible to exclude an extremely minute defect occurring *previously*, serial sections of some bladders showed definite implantation with no evidence of any ulceration whatsoever. If mucosal defects did occur in these cases they were so minute that for practical purposes the bladders must be considered intact. Aside from the presence of actual defects, the thickness of the bladder mucosa varied tremendously, from a thick pseudostratified layer in a contracted bladder to a thin, single layer of cuboidal cells in a dilated bladder (Fig. 7 and 8). Possibly these variations may have also played a part in the success or failure of implantation.

(b) *Kidney tumours.*—The renal tumours were an unexpected finding, and occurred commonly in all groups of animals. Their appearance and location was likewise very constant. In a moderately advanced stage they consisted of spherical masses which obliterated the recesses between cortex and medulla (Fig. 9 and 10). Their exact initial site was determined by studies of earlier lesions, with serial sections to locate the earliest foci. Early tumours never occurred within the renal parenchyma, nor did they appear on pelvic epithelium or on the central portion of the papillae. These surfaces, in the rat, are covered with high columnar and pseudostratified epithelium (Fig. 11). The deep recesses between cortex and pyramid, however, are normally lined by only a very thin flattened layer of cells more reminiscent of peritoneum than of urinary epithelium (Fig. 12). It was in these recesses that early lesions were always located, and serial sections of the earliest studies showed tumour cells in and immediately beneath the epithelium with formation of flat areas of replacement by tumour (Fig. 13, 14 and 15). Tumour cells were also found lying free within the recesses (Fig. 16). No evidence of pre-existing ulceration or inflammation was seen, and it seems almost certain that implantation occurred on intact epithelium. It may be suggested that the very thin lining in the recesses may be more susceptible to implantation than other areas, and resembles peritoneum in its susceptibility. Also, the deep recesses may represent a more "sheltered" region away from the main urinary flow, where cells are better able to survive.

### DISCUSSION

The initial studies, performed with the use of a fairly dilute tumour suspension and a relatively small inoculum, established clearly that traumatized epithelium is susceptible to implantation, but suggested that intact epithelium was resistant. Use of heavier suspensions and larger volumes produced a far higher incidence of implantation on normal bladder mucosa. We believe that implantation upon normal bladder mucosa has been demonstrated; or at least on mucosa which was essentially normal and had not been traumatized. If any defect is required for implantation to occur, it must be extremely minute in some cases, certainly no more than might be found in many human bladders in the presence of obstruction. It is difficult, also, to imagine any route except the urinary passages by which the tumour cells could have reached the bladder mucosa in the present experiments.

There is likewise no evidence that the renal tumours implanted exclusively on traumatized areas, since they occurred in crevices well sheltered from any external influence, and no ulceration or inflammation was seen in conjunction with the early tumours. The appearance and location of the early tumours, and the presence of tumour cells lying loose in the crevices immediately after injection by catheter, indicate that the tumour cells reached the kidneys by retrograde spread up the ureters. In other experiments we have found that trypan blue injected into the bladder in as little a volume as 0·25 c.c. can be seen to enter the ureters immediately. This reflux is not a surprising phenomenon; it has been observed with some human beings, especially in children, even with normal ureters.

It should be re-emphasized that the tumour used in the present experiment was not of urinary tract origin, and was far more malignant than the typical human tumour of renal pelvis, ureter or bladder. Also, the inoculum given was very large in the number of cells, probably much greater than would ever occur from exfoliation from a human tumour. On the other hand, only a single exposure occurred in the experiments, while human tumours may exfoliate into the lumen for years. It must also be borne in mind that implantation on minute defects occurred very readily; such defects might commonly exist in *apparently* normal human urinary tract, especially when infection or obstruction are present. These conditions often do exist when urinary tract tumours are present.

The present studies do not give a direct answer to the question of whether human urinary tract tumours spread by the urinary stream. They do indicate, however, *first,* that some tumour cells will survive in the urine long enough to implant successfully ; *second,* that they will implant very readily on very small mucosal defects, and under some circumstances, on intact epithelium.

SUMMARY

The ability of Walker 256 tumour to implant upon rat urinary mucosa has been studied by injections of cells into the ureter and urethra. Implantation occurred on a high proportion of bladders which had been traumatized, and upon some apparently intact bladders. Tumour cells also implanted in the kidneys, apparently secondary to retrograde passage up the ureters. The significance of these findings has been discussed.

This work was supported by a Grant-in-Aid from the National Cancer Institute of Canada.

We wish to thank Dr. J. M. Lederman for help with many portions of this investigation. We are grateful to Miss Audrey Backus and Mrs. Olga Neuendorff for technical assistance. Our thanks are due to Miss Martha Melville for performing the histological work, and to Miss Dea Neuendorff for photographic assistance.

### REFERENCES

Abeshouse, B. S.—(1956) *J. int. Coll. Surg.*, **25**, 117.
Albarran, J. and Imbert, L.—(1903) ' Les Tumeurs du Rein '. Paris (Masson et Cie). (Quoted by McDonald and Priestley.)
Allam, M. W., Lombard, L. S., Stubbs, E. L. and Shirer, J. F.—(1954) *Cancer Res.*, **14**, 734.
Baker, W. J. and Graf, E. C.—(1953) *J. Urol.*, **70**, 390.
Bronk, T. T. and Appel, M.—(1949) *Cancer Res.*, **9**, 228.
Ewing, J.—(1928) ' Neoplastic Diseases '. 3rd. Ed. Philadelphia (W. B. Saunders Company).
Furth, J.—(1946) *Amer. J. Path.*, **22**, 1101.
Hansemann, D.—(1890) *Virchows. Arch.*, **119**, 299.
Hershfield, E. S.—(1957) Thesis, University of Manitoba.
Hinman, F.—(1956) *J. Urol.*, **75**, 695.
Hovenanian, M. S.—(1950) *Ibid.*, **64**, 188.
Howell, R. D.—(1951) *Ibid.*, **66**, 561.
Hunt, V. C.—(1929) *Surg. Clin. N. Amer.*, **9**, 853.
Kaplan, J. H., McDonald, J. R. and Thompson, G. J.—(1951) *J. Urol.*, **66**, 792.
Kiefer, J. H.—(1953) *Ibid.*, **69**, 652.
MacAlpine, J. B.—(1947) *Brit. J. Surg.*, **35**, 113.—(1948) *Ibid.*, **36**, 164.
McDonald, D. F. and Lund, R. R.—(1954) *J. Urol.*, **71**, 560.
*Idem* and Thorson, T.—(1956) *Ibid.*, **75**, 690.
McDonald, J. R. and Priestley, J. T.—(1944) *Ibid.*, **51**, 245.
Mainland, D.—(1948) *Canad. J. Res.*, E26, 1.
Melicow, M. M.—(1952) *J. Urol.*, **68**, 261.
Norman, T. D. and McBroom, R. D.—(1957) *Surg. Forum*, **7**, 469.
Potompa, P. B. and Calhoun, C. W.—(1949) *West. J. Surg.*, **57**, 130.
Saltzstein, H. C. and Beaver, D. C.—(1940) *Arch. Surg.*, **40**, 949.
Stevens, W. E.—(1920) *J. Amer. med. Ass.*, **74**, 1576.
Wallace, A. C. and Hershfield, E. S.—(1957) *Proc. Amer. Ass. Cancer Res.*, **2**, 258.
Willis, R. A.—(1952) ' The Spread of Tumours in the Human Body '. 2nd Ed. London (Butterworth & Company).